# Has the COVID-19 Pandemic Impacted Healthcare Service Uptake at Hospitals in Addis Ababa?

**DOI:** 10.4314/ejhs.v31i4.2

**Published:** 2021-07

**Authors:** Tariku Shimels

**Affiliations:** 1 Saint Paul's Hospital Millennium Medical College, Addis Ababa, Ethiopia

**Keywords:** Addis Ababa, COVID-19, Ethiopia, health service utilization

## Abstract

**Background:**

The novel coronavirus has caused a profound impact on service utilization or delivery practices of health facilities globally. This study aims to evaluate the trend of health service utilization before and during the COVID-19 pandemic at selected hospitals in Addis Ababa, Ethiopia.

**Method:**

A facility-based cross-sectional study was conducted from 1^st^ through 30^th^ of August 2020 using a mixed-methods design. For the quantitative evaluation, ten months' time-series data, starting from September 2019 to July 2020 was retrieved from the HMIS unit of each hospital. Microsoft excel v.2010 was used to analyze quantitative data. Qualitative data was collected using a semi-structured key-informant interview guide and analyzed using QDA-minor software.

**Results:**

Twelve service delivery departments were included in the evaluation of each hospital. Of all, OPD, ART, VCT, PICT, and EPI services showed major disruption in both hospitals following the COVID-19 outbreak. Noticeable change was recorded in March and April for most units. Qualitative exploration showed multiple challenges namely; inadequate supply, poor infrastructure, low service utilization, staff workload, increased risk, poor job satisfaction of health professionals, and perception or attitude-related problems to be persistent at the hospitals.

**Conclusion:**

Main service delivery units of the hospitals, such as OPD, ART, VCT, PICT, and EPI have faced massive depression during the COVID-19 pandemic. The facilities had also encountered multifaceted challenges most from the internal environment. Immediate action should be in place to halt the negative impacts of the pandemic on the identified spots and challenges.

## Introduction

The coronavirus disease-2019 (COVID-19) pandemic caused worldwide turbulence affecting the health (1), economy (2, 3), social (4), and politics (5) of nations. As of January 4, 2021, the pandemic has infected 83,715,617 and killed 1,835,901 people globally (6). On this particular date too, Ethiopia accounted for 125,049 confirmed cases and 1,944 deaths. The trend of COVID-19 related cases and deaths continued to increase markedly, especially since July 2020, and the highest ever daily toll, until the submission date, was recorded on December 14, 2020 (1409 cases and 27 deaths).

The novel coronavirus has caused a profound impact on service utilization or delivery practices of health facilities globally. Nations even with the highest economy, technology, and human development index failed to withstand its catastrophic consequence. A study from Australia (7) showed that the pandemic has caused a significant reduction of private service utilization for manual therapies. As per a United States (US) report, shocks have also been reported from the health facilities side where professionals, such as Anesthesiologist faced to respond to critical cases and postpone elective procedures (8), and radiology units were anticipated to face 50% to 70% decreases (9). A significant drop in pediatric emergencies (10) and dental care services (11) has also been recorded during lockdowns in Germany.

Sub-Saharan Africa has been expected to be one of the high-risk regions with preexisting socio-economic predisposing and poor health infrastructure (12). Closure of borders, impaired supply chains, and prolonged lockdowns has created for lack of personal protective equipment (PPE), mental health problems, and substance abuse (13). An indirect effect of the COVID-19 pandemic on the region is markedly shown by the increased morbidity and mortality due to other tropical infections as malaria (14). In Ethiopia, the trend of health service uptake at public health facilities is limited even before the outbreak of COVID-19. Studies showed that patients may not visit health facilities for various reasons including; disease not requiring treatment, drugs bought from drug vendors, and visiting traditional healers (15). It was also suggested that multiple cultural, sociodemographic and facility-related determinants play a role in lower facility service utilization (16). In a study conducted in South Gondar, 24.6% of high school students utilized at least one reproductive health service, 55% used voluntary testing and counseling for HIV/AIDS and 51% utilized family planning services (17). A significant impact was noted on the service delivery practice of facilities or patients' uptake of most health services, such as treatment for Parkinson's disease (18), tuberculosis treatment (19), refill for follow-ups services (20), and an outpatient visit to hospitals (21).

Considering a wider spectrum of services might produce a comprehensive clue to understanding the effect of the pandemic. Besides, a time series-based trend analysis would present a precise illustration for the services most affected. The current study aims to evaluate the trend of health service utilization before and during the COVID-19 pandemic in selected public health facilities with a particular focus on hospitals in Addis Ababa, Ethiopia.

## Methods

**Study setting, design, and period**: A facility-based cross-sectional study was conducted at two hospitals namely; Saint Paul's Hospital Millennium Medical College (SPHMMC), and Ras Desta Damtew Memorial Hospital (RDDMH) both located in Addis Ababa, Ethiopia. Established in 1969, SPHMMC is one of the largest teaching hospitals under the federal ministry of health with 392 beds, an annual average of 200,000 patients, and a catchment population of over 5 million (22). Likely, RDDMH is a general hospital under the city government with a total number of 550 staff, 168 beds, and five different inpatient wards (23). The hospital also provides outpatient services, such as follow-up for chronic noncommunicable disease, family planning, immunization, gynecology, pediatrics, antiretroviral therapy (ART) among others. A mixed-methods design was employed from 1^st^ through 30^th^ of August 2020 to assess the trends of service utilization and challenges faced during the COVID-19 pandemic. Both the quantitative and qualitative data were collected concurrently.

**Population, sampling, data collection, and analysis**: For the quantitative trend analysis, a monthly patient load of various outpatient and inpatient service delivery units was considered. The units were selected purposively, with a high possibility of a chance to be affected by the COVID-19 pandemic in the respective hospitals. A ten months review period, starting from September 2019 to July 2020, was evaluated in this review. And, a structured data collection format was used to retrieve average monthly loads from the health management information system (HMIS) units of each hospital. Eight key informant interviews were undertaken with selected representatives from the outpatient, inpatient, disease prevention and health promotion, and pharmaceutical service departments. Inclusion of units and interviewees considered the potential encounter of compromised service delivery practice at major divisions of each hospital following the COVID-19 outbreak, a saturation of acquiring a new narration from the subsequent interviewees, and key informants' involvement in decision-making and supervisory roles before as well as during the pandemic. A semi-structured key informant interview (KII) guide was employed to collect the qualitative data. Quantitative data were analyzed using Microsoft Excel while qualitative data analysis minor (QDA-minor) was used for the qualitative part. Results were presented by figures and themes.

**Ethical approval**: Ethical approval was obtained from SPHMMC institutional review board (IRB) and the Addis Ababa regional health bureau (AARHB) regional IRB. A support letter was written to, and from the regional health, bureau to gain permission from one of the facilities. Data was analyzed in aggregates, and figures were presented in trend lines. No individual identification was included in the study.

## Results

**Figure 1:**
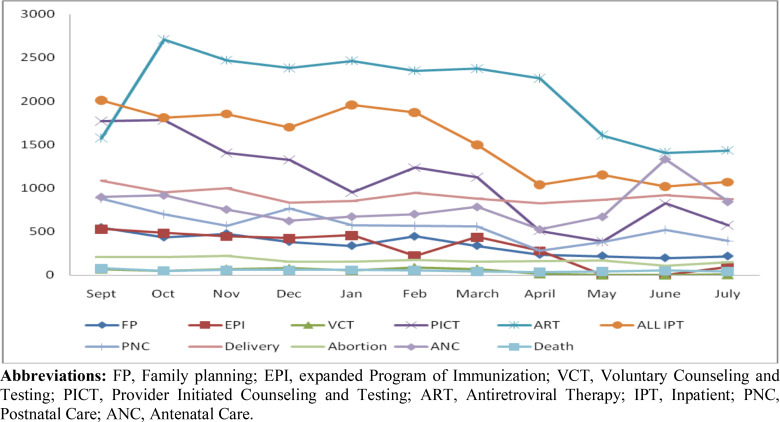
Patient flow at various service delivery units before and during the COVID-19 era in Addis Ababa, (hospital 1) **Abbreviations:** FP, Family planning; EPI, expanded Program of Immunization; VCT, Voluntary Counseling and Testing; PICT, Provider Initiated Counseling and Testing; ART, Antiretroviral Therapy; IPT, Inpatient; PNC, Postnatal Care; ANC, Antenatal Care.

**Patient flow trend by Service delivery points**: Inspecting the line graph at one of the hospitals presented below, a remarkable downshift of antiretroviral therapy (ART) services was noted as of April. Considering inpatient (IPT) services, a decrease in the number of admissions was noted in February compared to the same figure in the earlier month. The trough of this trend was recorded in April with a steadily consistent flow afterward. Provider initiated HIV counseling and testing (PICT) is one of the main outpatient services delivered in the hospital under constraints. Voluntarily counseling and testing (VCT) services also showed an unprecedented drop in and after April. This service has shown a dramatic contraction since the start of March with sustained fall in the subsequent months. An improvement was noted only in June still lower than the pre-COVID-19 era. The overall trend of ante-natal care (ANC) service utilization remained consistent until March. However, client visits fall in April and May. The service has shown a sudden rise in visits than earlier averages in June and July. In the same fashion, an expanded program of immunization (EPI) service utilization has been severely compromised by the pandemic. Whilst, earlier drop in the number of visits was noted in February, a significant fall was recorded after April. On the other hand, such services as postnatal care (PNC) except in April, the total number of beds, length of hospital stay, family planning (FP) utilization, death rates (inpatient) did not show a significant disturbance during the pandemic (Figure 1).

**Figure 2:**
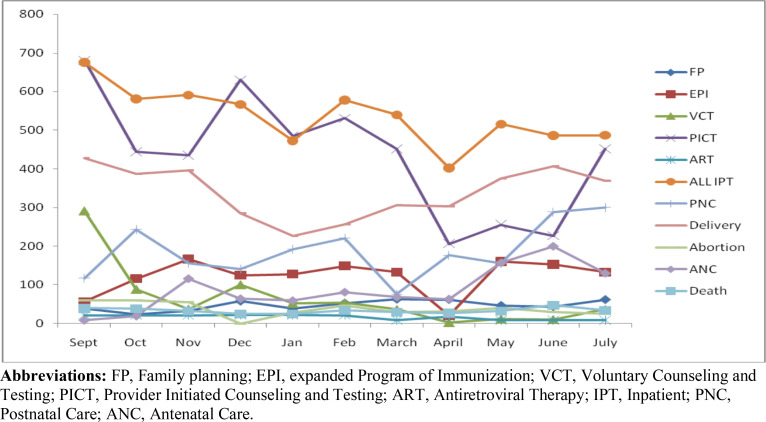
Patient flow at various service delivery units before and during the COVID-19 era in Addis Ababa, Ethiopia (hospital 2). **Abbreviations:** FP, Family planning; EPI, expanded Program of Immunization; VCT, Voluntary Counseling and Testing; PICT, Provider Initiated Counseling and Testing; ART, Antiretroviral Therapy; IPT, Inpatient; PNC, Postnatal Care; ANC, Antenatal Care.

The same number and types of parameters were observed in hospital 2 for the presence of any change during the outbreak. Accordingly, it was noted that many service units have experienced disruptions earlier times of the pandemic in the country though some, such as delivery, PICT, and EPI showed improvement in July. In April, EPI services, VCT, and PICT units faced severe disturbance in the number of services provided. All these services remained depressed during the following months until July. ART, PICT, and IPT services showed decrement since of February while EPI started to fall since of March (Figure 2).

**Figure 3:**
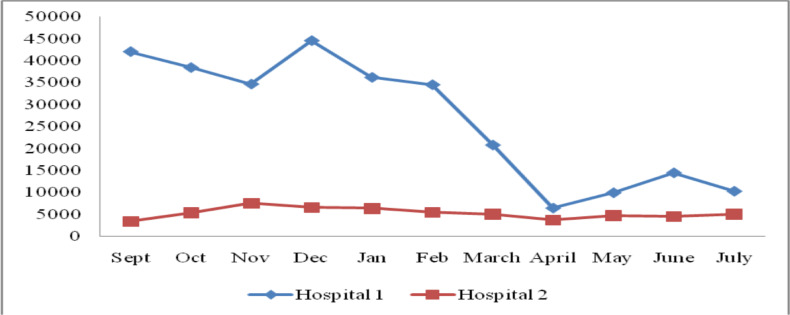
Trend of outpatient service visits before and during the COVID-19 pandemic at selected hospitals in Addis Ababa, Ethiopia

In a similar fashion, outpatient department (OPD) services per the respective hospitals were compared and presented in the figure below (Figure 3). Even though a high number of visits were recorded in November, it was found that significant falls happened in hospital 1 after January with the lowest hit in March. Nonetheless, OPD services did not exhibit a paramount disturbance throughout the review period at hospital 2 (Figure 3).

**Common challenges encountered, actions taken and ways forward**

**Challenges encountered**: The interviewees were asked what challenges the facility or the specific department had faced during the COVID-19 pandemic. Focus areas of interest included; outpatient services, inpatient services, disease prevention practices, and supply of medication and personal protective equipment (PPE). The challenges reported to have been experienced were classified and themed as supply related, infrastructure-related, service utilization, staff workload, increased risk, and poor satisfaction, and attitude related.

**Affected supply of healthcare inputs**: Among the challenges in this category, poor availability of personal protective equipment, such as N-95 masks, disposable masks, gloves, and alcohol were frequently mentioned. Similarly, shortage of medications as 40% dextrose, dexamethasone, aspirin (ASA) 81 mg, metformin, propylthiouracil (PTU), enalapril, and insulin was among the drugs with affected supply. The interviewees also raised that because the outbreak occurred in the middle of the budget year and it necessitated high demand for PPEs, responding to the problem as it needed was hardly possible.

**Inadequate infrastructure**: One of the challenges the facilities faced was reported to be a lack of adequate infrastructure. This mainly was reflected in limited space and facilities, such as waiting rooms, number of beds, facility compound, wastage disposal system (segregation), incinerators. It was also noted that increased patient load, high transmission rates, and risk of acquiring the virus among the staff posed additional challenges concerning inadequate infrastructure. Provision of health education was reported to be challenged due to lack of space (that accords with physical distancing rules), and lack of microphones. Some services as elective surgeries have been postponed due to the same reason.

**Low service utilization**: Not contrasted with overcrowding of services as a result of limited infrastructure and physical distancing rules, these challenges, on the other hand, appeared to mainly affect non-COVID-19 health care services. The interviewees emphasized that fear of acquiring the virus in general and framed information that the virus could (could not) be fatal for some groups have led to lower service uptake at the facilities. They stated that most of the units have been affected to deliver routing services as all resources and attention was drawn to prevent and manage the COVID-19. The fact that average monthly patient load figures were diminishing during the pandemic period (Figures 1 and 2) and the hospitals included in this study were designated as COVID-19 centers might contribute to the challenges purported.

**Staff workload, increased risk, and poor satisfaction**: Health professionals were the most to be challenged with the COVID-19 pandemic. The workload was created as a result of acquiring the infection as well as the government's rule to allow leave permission for those with advanced age or with comorbid conditions. This, in turn, has led to a cumulative effect on workload and frustration among the health care staff. They also mentioned that the impossibility to detect whether a suspect tested positive in shorter time intervals, their risk to get the virus was highly exacerbated. They added that other challenges (poor infrastructure, unknown suspects) have impacted transmission of the virus in hospital settings. Rotation of staff at some units (wards) was altered from the usual frequency, and that has increased their risk. Also, those working with emergency units, ambulances, and high contact areas faced fear and frustration. They stated that risk incentives were not either adequate or existent which made them unsatisfied at all. Further, the delayed reporting time of test results (as it was done only by the Ethiopian public health institute (EPHI)) and inability to coordinate these tasks (as they are external to the facilities), posed an additional risk, especially, to the healthcare staff. These all might have a significant effect on the compromised quality of services, less consultation time, and patient dissatisfaction.

**Attitude related challenges**: Perception on the effectiveness and persistent negligence to properly practice PPE use in the community was also a setback to prevent transmission of the virus key-informants stated. They also mentioned that people prefer either to hide or not disclose their close contacts due to fear of stigma and being relocated to quarantine centers. Health professionals, especially, those working in COVID-19 treatment centers and those who have been tested positive would face discrimination from neighbors and peers at the workplace.

**Actions taken to mitigate the challenges**: Interviewees were asked to mention actions taken by the facilities to mitigate challenges. Some units expressed that responses were either not taken at all during early times or were adequate to the scale of the problems. A multitude of efforts was, however, mentioned to have been implemented to curb the effects of the outbreak. These were broadly classified as a building of internal capacity, collaboration with other parties/facilities, and placement of multiple procurement options.

**Building internal capacity**: Actions under this theme included; delivery of training on prevention and control methods as well as management of COVID-19 cases to staff, an arrangement of additional rooms and beds, preparation of space for screening at entry points, isolation separate units/room or a building for COVID-19 suspects, prioritizing services based on the need of urgency and postponing some such as elective procedures, assigning extra work hours among staff to fill gaps, and availing of PPEs (masks, alcohol, and sanitizers) with locally made alternatives. Some have created and used mobile technology-based social media, such as a telegram to share timely information. One of the facilities was able to establish its testing unit later which reduced its long waiting time and increasing ques. The Millennium hall isolation center (an affiliate to SPHMMC) was also mentioned as an additional resource which at this time, has engaged to receive a full range of all COVID-19 suspects under the SPHMMC, leaving a room that the latter would turn to focus on all other healthcare services.

**Collaboration with other parties**: It was pointed out that various parties took part in the collaboration to fight against the COVID-19 pandemic, and update the information to the public. The bidirectional flow of information mainly steamed between the facilities and the federal ministry of health (FMOH) as well as the Ethiopian public health institute (EPHI) played a role in the synchronization of information on a daily number of cases, tests, and mortality rates. Collaboration also took place between specific facilities in referral linkages, waste segregation practices, training deliveries, and sharing of resources, such as PPEs and medicines. Some health centers and non-health affiliated organizations or buildings have been assigned as quarantine and isolation as well as treatment centers to facilitate the effectiveness of the campaign.

**Multiple procurement options**: To cope up with the sudden interruptions, the facilities have placed multiple procurement approaches. These included open tenders, restricted tender, and urgent proforma based on market availability and urgency of the needs. It was also raised that some supplies like PPEs were shared with other facilities, such as the millennium hall and Eka Kotebe Hospital. They also mentioned that though all efforts were not successful, organized committees or COVID-19 task force meetings used to take place for monitoring and evaluation of activities, including supplies.

**Further concerns and ways forward**: Key informants were asked to state what could be done to tackle the challenges encountered during the pandemic, and who should be involved as a stakeholder. Top management members and committees were suggested to monitor and recognize staff continuously. They also emphasized that some decisions and media coverages may not match with realities on the ground. In the hospital setting, key stakeholders should work to reduce to risk of transmission of the virus among health professionals and other patients. For example, delay in test results, and the transfer of patients towards before test result is released leads to the stated problem. Furthermore, the community should be aware that more people should not accompany suspected patients or any visit to hospitals.

It was also highlighted that sewerage systems and incinerators are either inadequate or not working during the study period that needs to be fixed. The federal ministry of health (FMOH), EPHI and the Addis Ababa regional health bureau were suggested to ensure proper resource allocation, prioritize interventions for action, and support downstream units. They said that communication channels should be synchronized and continuous supply of masks and gloves should be ensured. In line with this, they indicated that local manufacturers and suppliers must engage to work on highly demanded inputs and medicines such as Insulin. As one informant mentioned, the Ethiopian pharmaceuticals supply agency (EPSA) should also focus to avail essential medicines to the majority.

An additional concern that the informants put was the growing ignorance of non-COVID-19 cases in facilities. While they recommend that all coughing patients should get due attention for the possibility of suspect, other diseases need also be treated equally. If not managed properly or adhere to with treatment follow-ups, other diseases might potentially end with devastating outcomes as well. Therefore, an optimized approach to dealing with COVID-19 and non-COVID-19 cases would be highly encouraged in hospital settings.

## Discussion

The present study showed that most of the service delivery points in the respective hospitals have been affected by the COVID-19 outbreak since March. While the degree of alteration varied across departments, time frame, and level of specialty, massive changes in service drop were noted at ART, VCT, and EPI units of hospital 1. This is worth noting, however, that the massive fall of ART visits would largely be accounted to the implementation of the appointment spacing model (ASM) where a six months delivery of ART medications or services for stable HIV/AIDS patients has been started. Even though clients' full adherence to ART is required at all times, it remains to be difficult to figure out the actual number of cases with a full refill, on their ART or lost to follow up as trends happened to fall prominently after April. This, in turn, may call for a justification if the ASM practice has been intensified during the COVID-19 era.

As the first COVID-19 case was reported on the 13^th^ of March 2020 (24), most of the services considered showed a sudden collapse including ART and VCT. The link between ART and VCT services is linear such that the current protocol recommends for test and treatment initiation of any confirmed case. Profound diminishes in the number of people seeking VCT service might be an indication for the indirect effect of COVID-19 on the transmission of HIV and related complications. Reports based on a hypothetical model for sub-Saharan Africa showed that a 50% disruption for six months would cause for 6% increase in population-level mortality (25). And, the physical, emotional, and social well-being of ART clients could be greatly affected during this time leading to poor service uptake in this group (26).

On the other hand, time-series averages of the ten months service have been evaluated in the second hospital. Accordingly, EPI, VCT, and PICT units faced severe disturbance in the number of services provided during and after March. Despite one's expectation that only advanced or referral cases would appear to hospitals, the fact that it covers broader catchments with a high number of visits during the pre-COVID-19 era depicts a significant fall. It can also be deduced that some services, such as immunization might have either been shifted to local health centers or stopped due to fear of the COVID-19, but that remains to be determined. Yet, the sudden fall of many services during March is a prudent indication for large-scale failure in the health system associated with psychosocial disturbances (27), distancing rules, lockdowns, and uncertainties following the first COVID-19 case in the country (13,28).

A comparison of outpatient attendance and other service uptakes was made between the two hospitals considered. There was a considerable difference in terms of OPD visits whereby, hospital 2 showed a consistent flow throughout the review period. On the contrary, the same figure in hospital 1 revealed a sudden decrease in January compared to the previous month and a steadily sharp fall in March. This most likely attributed to the fact that hospital 1 was a teaching institution and COVID-19 treatment center whilst hospital 2 had only an isolation unit for COVID-19 suspects. A multitude of factors could have played a role in the reduced number of patient visits in the hospitals as key informant interviews showed. These may include but are not limited to; postponement of non-urgent cases, cancellation of elective surgeries, fear of acquiring the infection at hospitals, and extension of government rules (lockdowns, stay home rules, and physical distancing precautions).

Challenges faced amid the pandemic situation were explored in the present assessment. A similar finding has been reported in Australia (7), the US (8, 9), and Germany (10, 11). A source of further reason for patients' low confidence to attend the hospitals, even at times of acute illness, could be the availability of advanced healthcare infrastructure such as mechanical ventilators for patients with COVID-19. This was one of the highly narrated information, notably, at the beginning of the outbreak framing that countries in the sub-Saharan region would face devastating outcomes due to the pre-existing weak health system (12,29).

A qualitative exploration of the challenges faced following the COVID-19 outbreak was made to further understand the problem from the perspective of health professionals. The key informants raised a multitude of problems prominently hindering their service delivery practices. These included; supply-related, poor pre-existing infrastructure, low service utilization of patients, staff workload, increased risk of health professionals to the virus, poor job satisfaction of health professionals, and attitude-related challenges. Some of the challenges reported are interrelated to each other such that either solving or failing to solve one may imply the other. For example, attitude-related challenges have been reported to affect the perception of disease causation (30), severity (31), and prevention measures (32). It has also been reported that patients or health professionals faced stigma from neighbors or colleagues in being a survivor or a suspect to the coronavirus.

Poor infrastructure such as lack of facility space and medical equipment may also worsen poor availability of medications and PPEs, staff work overload, increased risk to acquire the infection, low OPD attendance, and poor service satisfaction. The fact that the outbreak occurred at mid physical year along with an exhausted and limited budget to some programs might have militated the situation. In fact, within the limit of efforts undertaken so far, the facilities have shown to build internal capacity, collaboration with stakeholders, and placement of multiple procurement options. In line with this, the informants also mentioned that the implementation of ‘stay home rules for COVID-19 suspects’ has posed a negative influence on patients with old age and comorbid conditions as there is no adequate staff, facility, or medical equipment that can be mobilized to home-based care.

Overall, the present study showed that antiretroviral therapy, outpatient visits, immunization, voluntary counseling and testing, and provider-initiated HIV counseling and testing have been severely affected during the COVID-19 pandemic. Almost all service units encountered a significant fall in services in March and April. Key informants also showed that challenges including; inadequate supply of health service delivery inputs, poor pre-existing infrastructure, low service utilization of patients, staff workload, increased risk of health professionals to the virus, poor job satisfaction of health professionals, and wrong perception or attitude were dominant during the outbreak. The results are anticipated to inform health professionals, hospital management, politicians, and health authorities across the country. The study, however, is limited to only two hospitals which can affect the generalizability of context-specific measures. A prospective evaluation with more facilities included is warranted to uncover all impacts of the pandemic.
